# Luminal flow induces NADPH oxidase 4 translocation to the nuclei of thick ascending limbs

**DOI:** 10.14814/phy2.12724

**Published:** 2016-03-31

**Authors:** Fara Saez, Nancy J. Hong, Jeffrey L. Garvin

**Affiliations:** ^1^ Department of Physiology and BiophysicsSchool of MedicineCase Western Reserve UniversityClevelandOhio

**Keywords:** Gene transcription, hypertension, Nox 4, redox signaling, superoxide

## Abstract

Superoxide (O_2_^−^) exerts its physiological actions in part by causing changes in gene transcription. In thick ascending limbs flow‐induced O_2_^−^ production is mediated by NADPH oxidase 4 (Nox4) and is dependent on protein kinase C (PKC). Polymerase delta interacting protein 2 (Poldip2) increases Nox4 activity, but it is not known whether Nox4 translocates to the nucleus and whether Poldip2 participates in this process. We hypothesized that luminal flow causes Nox4 translocation to the nuclei of thick ascending limbs in a PKC‐dependent process facilitated by Poldip2. To test our hypothesis, we studied the subcellular localization of Nox4 and Poldip2 using confocal microscopy and O_2_^−^ production in the absence and presence of luminal flow. Luminal flow increased the ratio of nuclear to cytoplasmic intensity of Nox4 (N/C) from 0.3 ± 0.1 to 0.7 ± 0.1 (*P* < 0.01) and O_2_^−^ production from 89 ± 15 to 231 ± 16 AU/s (*P* < 0.001). In the presence of flow PKC inhibition reduced N/C from 0.5 ± 0.1 to 0.2 ± 0.1 (*P* < 0.01). Flow‐induced O_2_^−^ production was also blocked (flow: 142 ± 20 AU/s; flow plus PKC inhibition 26 ± 12 AU/s; *P* < 0.01). The cytoskeleton disruptor cytochalasin D (1 *μ*mol/L) decreased flow‐induced Nox4 translocation by 0.3 ± 0.01 (*P* < 0.01); however, it did not reduce flow‐induced O_2_^−^. Flow did not alter Poldip2 localization. We conclude that: (1) luminal flow elicits Nox4 translocation to the nucleus in a PKC‐ and cytoskeleton‐dependent process; (2) Nox4 activation occurs before translocation; and (3) Poldip2 is not involved in Nox4 nuclear translocation. Flow‐induced Nox4 translocation to the nucleus may play a role in O_2_^−^‐dependent changes in thick ascending limbs.

## Introduction

Superoxide (O_2_^−^) is a reactive oxygen species that regulates renal function (Zou et al. 2001; Wilcox 2002). Excessive production causes salt retention (Majid and Nishiyama 2002), renal damage (Hisaki et al. 2005; Manning et al. 2005), and hypertension (Hisaki et al. 2005; Kopkan and Majid 2005). NADPH oxidase 4 (Nox4) is the main source of thick ascending limb, and thus, medullary O_2_^−^ (Hong and Garvin 2012). Superoxide production in this nephron segment is stimulated in part by increases in luminal flow (Hong and Garvin 2007, 2012), which may be caused by a high‐salt diet (Mozaffari et al. 1991), the early stages of diabetes (Pollock et al. 1991) and hypertension (Baer et al. 1978; DiBona and Rios 1978). Flow‐induced increases in O_2_^−^ production can be blocked by inhibiting PKC or expressing kinase‐dead dominant‐negative PKC*α* mutants (Hong et al. 2010).

Chronically elevated O_2_^−^ in the thick ascending limb causes changes in transcription and translation of a number of proteins (Riazi et al. 2009; Roson et al. 2011). The mechanisms by which O_2_^−^ changes gene transcription are not completely understood. Since O_2_^−^'s negative charge limits its membrane permeability (Lynch and Fridovich 1978; Salvador et al. 2001) and it is highly reactive with many molecules including water, the actions of O_2_^−^ are likely to be localized to the area in which it is produced. As a result, transcriptional changes elicited by Nox4‐derived O_2_^−^ in thick ascending limbs are likely to occur either: (1) directly in the nucleus via nuclear Nox4 itself (Liu et al. 2010); or (2) indirectly in the cytoplasm by affecting redox‐sensitive cytoplasmic transcription factors which subsequently translocate to the nucleus (Brewer et al. 2011) rather than by O_2_^−^ diffusing into the nucleus through the nuclear envelope.

Translocation of Nox4 from the cytoplasm to the nucleus may be facilitated by binding partners such as polymerase delta interacting protein 2 (Poldip2). Poldip2 interacts with Nox4 (Lyle et al. 2009) and is thought to translocate to the nucleus when activated (Klaile et al. 2007; Wong et al. 2013). It increases reactive oxygen species production in vascular smooth muscle cells and drives subcellular Nox4 localization by interacting with the actin cytoskeleton (Lyle et al. 2009).

Thus, we hypothesize that increases in luminal flow cause PKC‐dependent translocation of Nox4 to the nucleus of thick ascending limb cells, and that Poldip2 facilitates this process.

## Materials and Methods

### Animals

Male Sprague–Dawley rats (Charles River Breeding Laboratories, Wilmington, MA) were fed a diet containing 0.22% sodium and 1.1% potassium (Purina, Richmond, IN) for at least 4 days before the experiments. All animal protocols were previously reviewed and approved by the Institutional Animal Care and Use Committee of Case Western Reserve University following the National Institutes of Health *Guide for the Care and Use of Laboratory Animals*.

### Chemicals and solutions

The composition of the physiological saline used to perfuse and bathe thick ascending limbs was (in mmol/L) 130 NaCl, 2.5 NaH_2_PO_4_, 4 KCl, 1.2 MgSO_4_, 6 l‐alanine, 1 trisodium citrate, 5.5 glucose, 2 calcium dilactate, and 10 HEPES, pH 7.4 at 37°C. All solutions were adjusted to 290 ± 3 mOsmol/kg H_2_O as measured by vapor pressure osmometry. Dihydroethidium was purchased from Life technologies (Eugene, OR), staurosporine was from Roche Applied Science (Indianapolis, IN), Gö 6976 was from Enzo Life Sciences (Farmingdale, NY) and cytochalasin D was purchased from Sigma (St. Louis, MO). The goat polyclonal antibody against the amino terminus of Nox4 (N‐Nox4; sc‐21860) and the goat polyclonal antibody against Poldip2 (sc‐82998) were obtained from Santa Cruz Biotechnology (Santa Cruz, CA). The rabbit monoclonal antibody against the carboxyl terminus of Nox4 (C‐Nox4; ab109225) and the rabbit polyclonal antibody against Histone 3 (ab62642) were from Abcam (Cambridge, UK). Alexa Fluor secondary antibodies and the DNA marker POPO3 were purchased from Life technologies (Eugene, OR).

### Isolation and perfusion of thick ascending limbs

Rats weighing 120–150 g were anesthetized with ketamine and xylazine (100 and 20 mg/kg body wt., respectively). The abdominal cavity was opened and the left kidney was bathed in ice‐cold saline and removed. Coronal sections were placed in physiological saline and medullary thick ascending limbs were dissected from the outer medulla under a stereomicroscope at 4–10°C. Tubules ranging from 0.5 to 1.0 mm were transferred to a temperature‐regulated chamber (37 ± 1°C) and perfused using concentric glass pipettes as described previously (Garvin et al. 1988). The luminal perfusion rate was either 0 (no flow) or about 10 nL/min using gravity. The flow rate of the basolateral bath was 0.5 mL/min.

### Immunodetection of Nox4 and Poldip2 in isolated thick ascending limbs using confocal microscopy

Tubules were either perfused or held on the pipets in the absence of flow for 20 min. They were then fixed for 15 min with 4% paraformaldehyde in phosphate‐buffered saline (PBS), pH 7.4, on the stage of a confocal microscope. Fixed cells were permeabilized with Triton^®^ X‐100 (0.5%) and blocked for 30 min with 3% bovine serum albumin (BSA) in PBS followed by a 2‐h incubation with primary antibodies against Nox4 and Poldip2 diluted in 1% BSA in tris‐buffered saline‐tween (TBS‐T). Nox4 was detected using two different commercially available antibodies, as described above. Tubules were washed with PBS in the bath for 5 min and then incubated for 30 min with suitable secondary Alexa Fluor antibodies. All antibodies were diluted 1:1000 in 1% BSA in TBS‐T. Cells were then washed for 5 min with PBS in the bath. Images were acquired using confocal microscopy (Visitech, Sunderland, UK). Nuclei were counterstained with either POPO3 or with an antibody against Histone 3. For experiments using the PKC inhibitor staurosporine (10 nmol/L) or the cytoskeleton disruptor cytochalasin D (1 *μ*mol/L), tubules were perfused and the drug or vehicle (dimethyl sulfoxide (DMSO) 0.001%) was added to the bath. After 30 min, Nox4 and/or Poldip2 were detected via immunofluorescence as described above.

### Image analysis

Images were acquired with a 100× objective and observed at 1750× linear magnification. Cells were only analyzed when the nucleus was showing its maximum profile. Two cells were evaluated per tubule. Within each cell, two regions of interest (ROI) were selected: nuclear and cytoplasmic. Mean gray intensities within these regions were measured using the open‐source National Institutes of Health (NIH) software ImageJ (imagej.nih.gov). Results are expressed as the ratio of intensities in the nuclear and cytoplasmic ROIs (N/C intensity). For colocalization analysis, the ImageJ plugin JACoP (http://rsb.info.nih.gov/ij/plugins/track/jacop.html) was used to estimate Pearson coefficients in nucleus and cytoplasm from perfused thick ascending limbs that were stained with both, C‐Nox4 and Poldip2 antibodies.

### Measurement of O_2_^−^ using dihydroethidium

Isolated thick ascending limbs were loaded with 10 *μ*mol/L dihydroethidium in physiological saline for 15 min and then washed in dye‐free solution for 20 min. Oxyethidium and dihydroethidium were excited at 490 and 365 nm, respectively. Fluorescence emitted between 520 and 600 nm (oxyethidium) and 400–450 nm (dihydroethidium) was imaged digitally with an image intensifier connected to a CCD camera and recorded with a Metafluor imaging system (Universal Imaging, West Chester, PA). Fluorescence from ROI was measured every 5 sec for 12 measurements. Measurements were obtained as follows: after 20 min washing, the no flow period (1 min) was acquired and immediately after, flow was started again and data for the flow period (1 min) recorded. Regression analysis of the fluorescence ratios for each measurement period was performed and differences in slopes were evaluated using the first six measurements, taken as the initial rate of stimulation. An increased rate of change in the oxyethidium/dihydroethidium fluorescence ratio is a measure of increased O_2_^−^ production. We refer to O_2_^−^ measurements in this study as O_2_^−^ production and the results are expressed in arbitrary units per second (AU/sec). For experiments in which the PKC inhibitor Gö 6976 (10 nmol/L) or cytochalasin D (1 *μ*mol/L) were used, the drugs were added to the bath during the wash period and were present throughout the experiment.

### Statistical analysis

Results are expressed as means ± SE. Two‐tailed unpaired Student's *t*‐test was used to compare means between different tubules (immunofluorescence studies and O_2_^−^ experiments in which the effect of inhibitors were studied). Two‐tailed paired Student's *t*‐test was used for the O_2_^−^ production experiments in which period 1 (no flow) is compared to period 2 (flow) within the same tubule. *P *<* *0.05 was considered significant.

## Results

**Figure 1:**
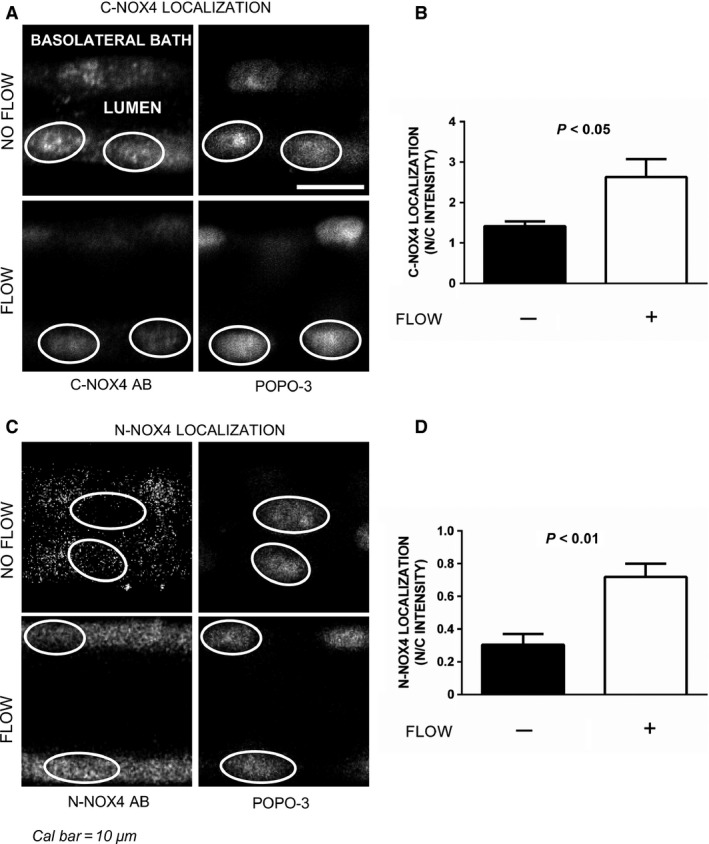
Effect of luminal flow on Nox4 localization and activation in thick ascending limbs. Panels A and B: Effect of luminal flow on Nox4 localization in thick ascending limbs using an antibody against the carboxyl terminus of Nox4 (C‐Nox4); (A) Representative immunofluorescent confocal images showing C‐Nox4 staining (left panel) and same nuclei counterstained with POPO3 (right panel). (B) Quantification using the ratio between nuclear and cytoplasmic intensities (N/C intensity; *n *=* *5 and 6, for no flow vs. flow, respectively). Panels C and D: Effect of luminal flow on Nox4 localization in thick ascending limbs using an antibody against the amino terminus of Nox4 (N‐Nox4); (C) Representative immunofluorescent confocal images showing N‐Nox4 staining (left panel) and same nuclei counterstained with POPO3 (right panel). (D) Quantification using the ratio between nuclear and cytoplasmic intensities (N/C intensity; *n *=* *6 for each group). Nuclei are outlined for better identification.

To begin to test our hypothesis, we first studied whether increasing luminal flow caused Nox4 translocation to the nucleus using the C‐Nox4 antibody (Fig. 1A and B). In the absence of luminal flow, Nox4 appeared evenly distributed in both the cytoplasm and nucleus (Fig. 1A). Thus, the ratio of intensities in the nuclear and cytoplasmic regions (N/C intensity) was near unity (Fig. 1B). When tubules were subjected to flow, the N/C intensity increased from 1.4 ± 0.1 to 2.6 ± 0.4, an 86% increase (*P < *0.05; *n *=* *5 and 6, respectively).

We next repeated these experiments using the antibody directed against the amino terminus (Fig. 1C and D). With the N‐Nox4 antibody we found that in the absence of luminal flow, cytoplasmic staining was intense while that of the nuclear region was faint, as shown by the representative confocal images in Figure 1C and the data represented in Figure 1D (N/C intensity 0.3 ± 0.1). When tubules were subjected to flow, the N/C intensity increased to 0.7 ± 0.1, a 133% increase (*P *<* *0.01; *n *=* *6 for each group). Taken together with the results depicted in Figure 1A and B, these data show that increasing luminal flow causes Nox4 to translocate to the nucleus.

**Figure 2:**
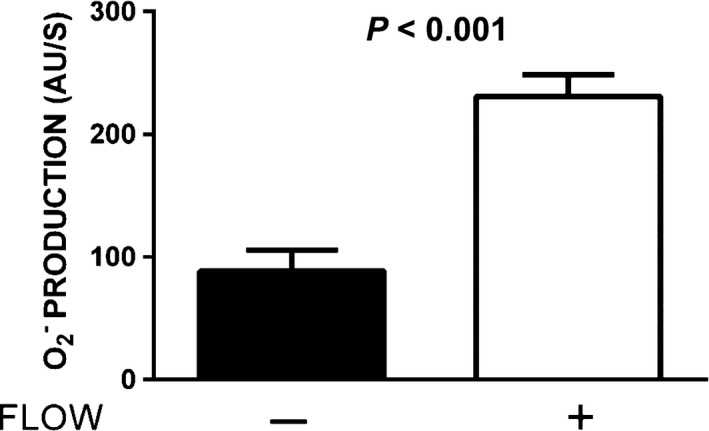
Effect of luminal flow on O_2_^−^ production by thick ascending limbs (*n *=* *6).

To study the relationship between Nox4 localization and flow‐induced O_2_^−^ production, we first needed to demonstrate that increasing luminal flow‐stimulated O_2_^−^ production, and then we used different inhibitors to test whether the two processes were interdependent. Figure 2 shows that raising luminal flow from 0 to about 10 nL/min stimulated O_2_^−^ production from 89 ± 15 to 231 ± 16 AU/sec (*P *<* *0.001; *n *=* *6). These data show that flow stimulates O_2_^−^ production.

**Figure 3:**
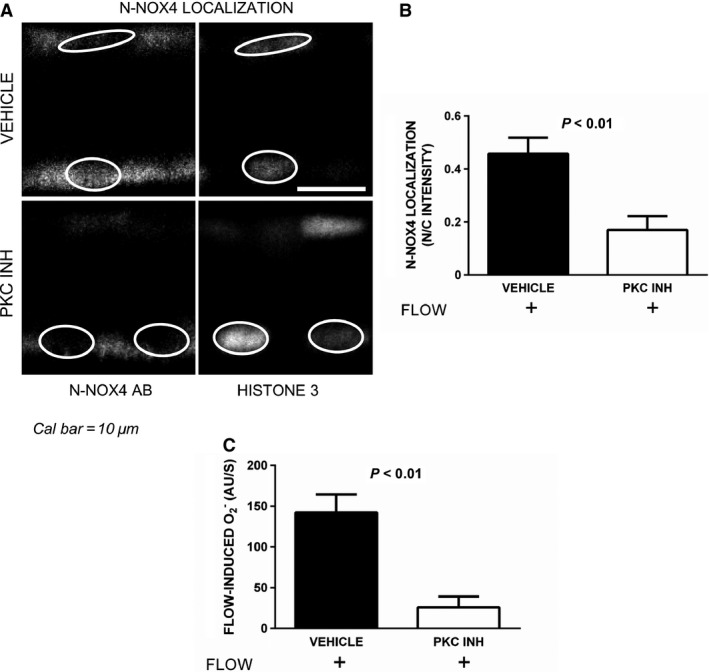
Effect of PKC inhibition (PKC INH) on Nox4 localization and O_2_^−^ production in the presence of flow in thick ascending limbs. Panels A and B: Nox4 localization expressed as the ratio between nuclear and cytoplasmic intensities using the N‐Nox4 antibody. (A) Representative immunofluorescent confocal images showing N‐Nox4 staining (left panel) and same nuclei counterstained with Histone 3 (right panel). (B) Quantification using the ratio between nuclear and cytoplasmic intensities (N/C intensity; *n *=* *4 and *n *=* *5, for vehicle vs. PKC inhibitor, respectively). Nuclei are outlined for better identification. Panel C: Effect of PKC inhibition on flow‐induced O_2_^−^ production (*n *=* *6 for each group).

Because PKC mediates flow‐stimulated O_2_^−^ production in thick ascending limbs (Hong et al. 2010), we hypothesized that PKC plays a role in flow‐induced recruitment of Nox4 to the nucleus. Figure 3A displays representative confocal images showing the effects of inhibiting PKC with staurosporine on flow‐induced Nox4 translocation. In these experiments, the N/C intensity was 0.5 ± 0.1 in the presence of luminal flow (Figure 3B, *n *=* *4). In contrast, when PKC was inhibited the N/C intensity in the presence of flow was only 0.2 ± 0.1 (Figure 3B, *n *=* *5; *P *<* *0.01). Thus, the N/C intensity was 150% greater with flow alone than with flow plus PKC inhibition.

Because PKC inhibition prevented flow‐induced Nox4 translocation, we next tested whether it blocks O_2_^−^ production (Figure 3C). Flow‐induced O_2_^−^ production in isolated thick ascending limbs was 142 ± 20 AU/sec in controls, but only 26 ± 12 AU/sec when PKC was inhibited with Gö 6976 (*P *<* *0.01, *n *=* *6 and *n *=* *5 for each group, respectively). These results show that both Nox4 redistribution to the nucleus and activation in response to luminal flow depends on PKC activity.

**Figure 4:**
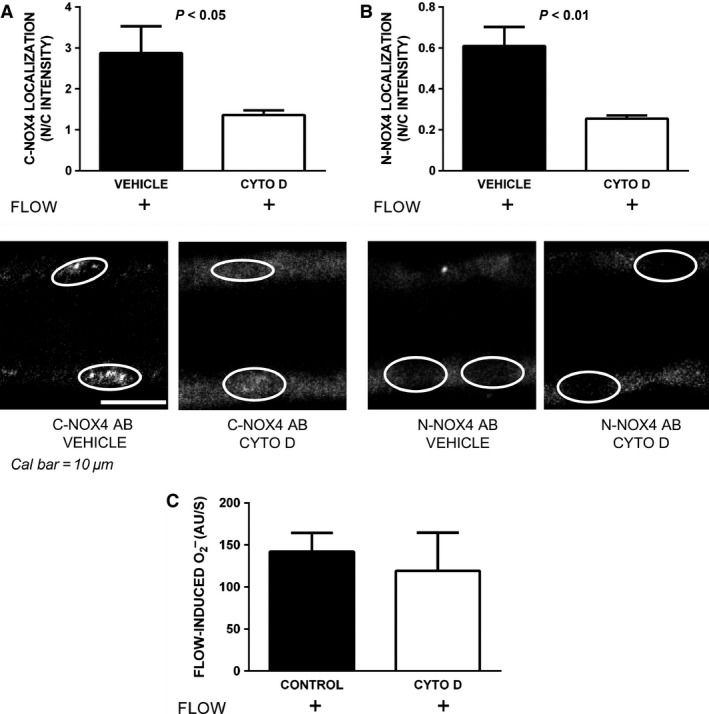
Effect of disrupting the cytoskeleton with cytochalasin D (CYTO D) on Nox4 localization and O_2_^−^ production in the presence of flow in thick ascending limbs. Panel A: Top: Nox4 localization expressed as the ratio between nuclear and cytoplasmic intensities (N/C intensity) using the C‐Nox4 antibody (*n *=* *3 and 4, for vehicle vs. cytochalasin D, respectively), bottom: representative immunofluorescent confocal images of tubules treated either with vehicle (left panel) or CYTO D (right panel). Panel B: Top: Nox4 localization expressed as the ratio between nuclear and cytoplasmic intensities (N/C intensity) using the N‐Nox4 antibody (*n *=* *5 for each group), bottom: representative immunofluorescent confocal images of tubules treated either with vehicle (left panel) or CYTO D (right panel). Panel C**:** Effect of cytochalasin D on flow‐induced O_2_^−^ production (*n *=* *6 and 7, for control vs. cytochalasin D, respectively).

Although the proteins involved in luminal flow‐induced Nox4 translocation are not known, it is likely that an intact actin cytoskeleton is required. Thus, we next tested whether a cytoskeletal disruptor, cytochalasin D, would prevent flow‐induced Nox4 translocation to the nucleus (Fig. 4A and B). In the presence of vehicle, N/C intensity was 2.9 ± 0.5 in the presence of flow as measured using the C‐Nox4 antibody (*n *=* *3). However, it was only 1.4 ± 0.1 in the presence of 1 *μ*mol/L cytochalasin D (*n *=* *4; *P *<* *0.05; Fig. 4A). Thus, the N/C intensity was 107% greater with flow alone than with flow plus cytochalasin D. Similarly, the N/C intensity was 0.6 ± 0.1 in controls in the presence of luminal flow and 0.3 ± 0.01 in cytochalasin D‐treated tubules when the N‐Nox4 antibody was used (*P *<* *0.01, *n *=* *5 for each group; Fig. 4B). These data show that an intact cytoskeleton is required for Nox4 translocation to the nucleus.

Because cytochalasin D inhibited translocation of Nox4, we next tested whether it would have a similar effect on flow‐induced O_2_^−^ production in isolated thick ascending limbs. Figure 4C shows that in the presence of cytochalasin D, O_2_^−^ production increased by 119 ± 42 AU/sec when luminal flow was increased (*n *=* *7), not significantly different from that of the control group (*n *=* *6). These results suggest that flow‐induced increases in Nox4 activation, and thus O_2_^−^ production, occur prior to translocation to the nucleus.

**Figure 5:**
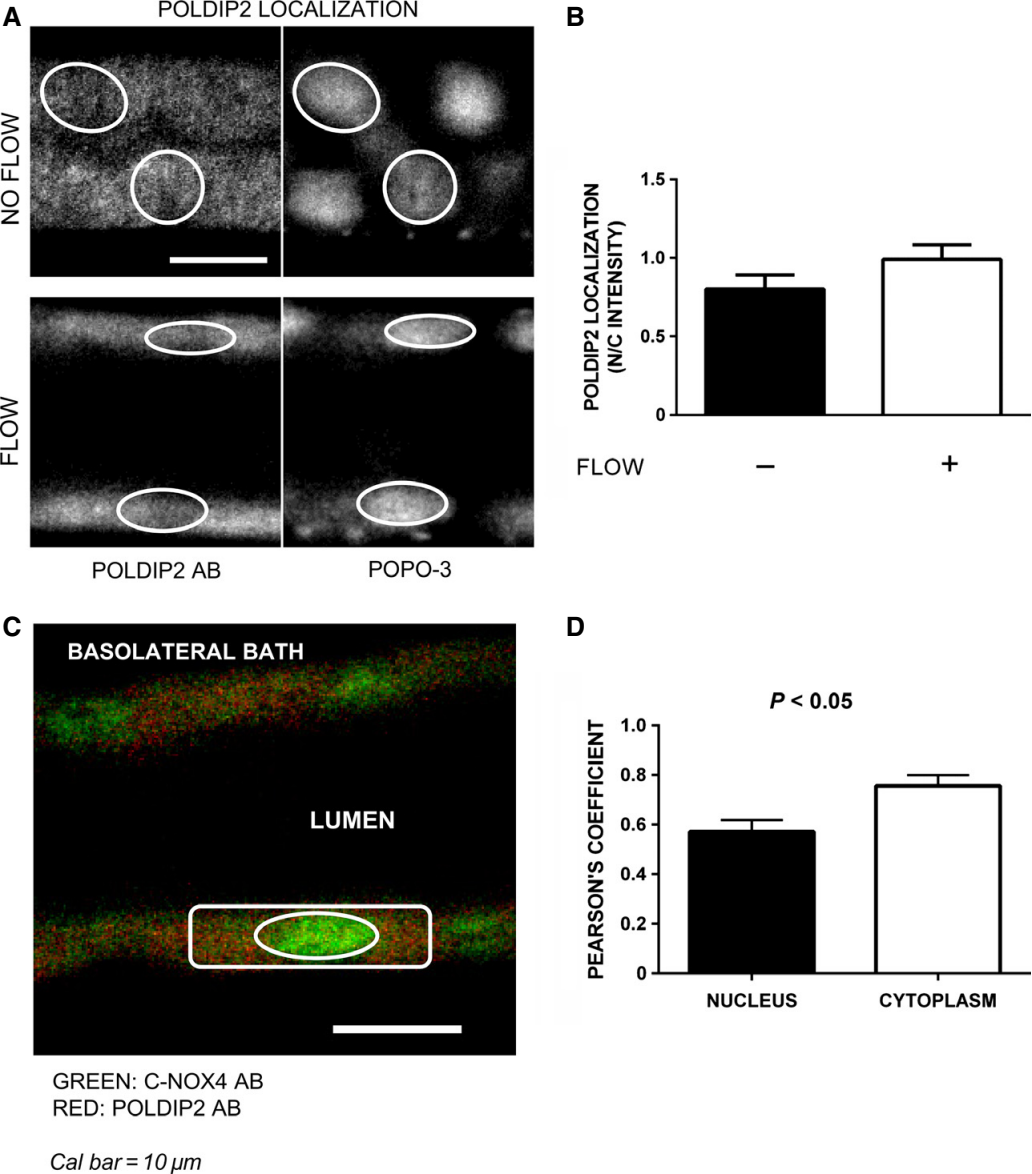
Effect of luminal flow on Poldip2 localization in thick ascending limbs. Panel A: Representative immunofluorescent confocal images showing Poldip2 staining (left panel) and same nuclei counterstained with POPO3 (right panel). Panel B: Effect of flow on Poldip2 localization expressed as the ratio between nuclear to cytoplasmic intensities (N/C intensities; *n *=* *4 and 6, for no flow vs. flow, respectively). Panel C and D: Colocalization data from perfused thick ascending limbs stained for C‐Nox4 and Poldip2. C: Representative confocal image of a perfused tubule stained with both antibodies. Nucleus and cytoplasm are outlined for better identification. D: Colocalization data expressed as Pearson's coefficient (*n *=* *3)

Poldip2 has been shown to interact with Nox4 and regulate its activity in vascular smooth muscle cells (Lyle et al. 2009). Since this protein may be important for Nox4 activation and the regulation of O_2_^−^ production in thick ascending limbs, we next examined the effect of flow on its intracellular localization (Fig. 5A and B). Figure 5A displays representative confocal images of nonperfused and perfused tubules stained for Poldip2. In the absence of flow the Poldip2 N/C intensity was 0.8 ± 0.1 (*n *=* *4). In tubules exposed to luminal flow it was 1.0 ± 0.1 (*n *=* *6), not significantly different. When colocalization studies of Nox4 and Poldip2 were carried out in perfused thick ascending limbs, Pearson's coefficient was significantly higher in the cytoplasmic compared to the nuclear region (0.8 ± 0.04 vs. 0.6 ± 0.04, *P *<* *0.05, Fig. 5C and D), showing a similar compartmentalization of Nox4 and Poldip2 in the cytoplasmic region but not in the nucleus. These results suggest that Nox4 translocation to the nucleus is independent from Poldip2.

## Discussion

This study shows that luminal flow in thick ascending limbs leads to Nox4 recruitment to the nuclear region of thick ascending limb cells and an increase in O_2_^−^ production. Both flow‐induced translocation and O_2_^−^ production are blocked by inhibiting PKC, but only translocation is prevented by disrupting the cytoskeleton. Increasing luminal flow did not cause Poldip2 to translocate to the nucleus.

Chronically elevated O_2_^−^ causes changes in protein expression in several cell types (Riazi et al. 2009; Vendrov et al. 2010; Roson et al. 2011; New et al. 2012) including thick ascending limbs (Riazi et al. 2009; Roson et al. 2011). Many of these changes are likely due to up or downregulation of gene transcription (Liu et al. 2010; Vendrov et al. 2010; Brewer et al. 2011; Brigelius‐Flohe and Flohe 2011). Nox4 could alter transcription by translocating to the nucleus and producing O_2_^−^ there; producing O_2_^−^ in the cytoplasm or other non‐nuclear compartments which then diffuses to the nucleus; and/or generating O_2_^−^ outside the nucleus which then affects redox‐sensitive transcription factors that subsequently move to the nucleus. Our data show that an antibody against the carboxyl terminus of Nox4 showed higher fluorescence in the nucleus in thick ascending limbs exposed to luminal flow than those that were not. We also found that when we used an antibody that recognizes the amino terminus it also showed that flow induces Nox4 translocation to the nucleus. These data show that Nox4 translocates to the nucleus in response to flow. They may further suggest that one mechanism by which flow‐stimulated O_2_^−^ alters gene transcription is via Nox4‐dependent nuclear translocation and superoxide generation.

Immunofluorescence as a method to quantify subcellular localization and translocation is affected by many variables including excitation intensity, sensitivity of the camera, antibody binding, and selectivity of the antibodies used. By normalizing the nuclear fluorescence to the cytoplasmic fluorescence, we eliminated many of these confounding variables. Furthermore, since we used two different Nox4 antibodies and found similar results, our data are not likely significantly influenced by nonspecific binding. Use of immunofluorescence after perfusing and fixing the tubule is the most appropriate method to determine translocation elicited by luminal flow. Other approaches such as Western blotting of nuclear fractions are not appropriate. To perform Western blots, one would have to use cultured thick ascending limbs cells, and there is no guarantee that cultured cells would behave in a manner similar to native cells. We have also demonstrated that the effects of flow last roughly for 15 min (Garvin and Hong 2008). By the time nuclear fractions were prepared, the effects elicited by flow most probably would be lost. Finally, subcellular fractionation never yields absolutely pure fractions. Any contamination of the nuclear fraction with cytoplasm would eliminate our ability to detect flow‐induced differences in the amount of Nox4 in the nuclear fraction due to the relative amounts in each compartment.

Our data show that Nox4 is widely distributed within thick ascending limb cells. This finding is supported by reports showing that it has been found in mitochondria (Block et al. 2009), endoplasmic reticulum (Lee et al. 2010; Wu et al. 2010), plasma membrane (Xi et al. 2013), and the nucleus of several cell types (Kuroda et al. 2005; Lyle et al. 2009; Lee et al. 2010; Matsushima et al. 2013). Our finding that Nox4 localizes to the nucleus of thick ascending limbs is the first such report for renal epithelial cells. Nox4 has been shown to localize to the nucleus of other cell types including vascular muscle cells (Lyle et al. 2009; Anilkumar et al. 2013), cardiomyocytes (Matsushima et al. 2013), hepatocytes (Spencer et al. 2011), and endothelial cells (Kuroda et al. 2005). Although Nox4 contains two nuclear localization signals (Pendergrass et al. 2009), the protein is also predicted to have six transmembrane domains. As a result, the question remains as to how it localizes to the nucleus.

Nox4 has several splice variants (Goyal et al. 2005). Recently, the presence of a Nox4 splice variant has been described in the nucleus of several cell types and its overexpression increased phosphorylation of extracellular‐signal‐regulated kinase1/2 and the nuclear transcription factor Elk‐1. Overexpression of this splice variant also induced DNA damage (Anilkumar et al. 2013). One possible explanation for the nuclear localization of Nox4 is that the soluble splice variant lacking the transmembrane regions is the protein that resides there (Anilkumar et al. 2013). However, our results from experiments in which antibodies were used against the carboxyl and amino termini suggest that the translocation of Nox4 splice variants is a general phenomenon. Thus, the question of which splice variant(s) reside in the nucleus and how they get there remains unanswered.

We studied Nox4 localization only after exposing tubules to 20 min of luminal flow. It is therefore possible that Nox4 translocation is a relatively slow process taking as long as 20 min. Consequently, our results could be due to translocation of Nox4 from other compartments to the nucleus or de novo synthesis of Nox4 protein which directly enters the nucleus. Several studies have shown that Nox4 protein expression can increase in as little as 5 min (New et al. 2012; Eid et al. 2013; Lee et al. 2013). However, de novo synthesis of Nox4 as an explanation for our data seems unlikely. We found no evidence of flow inducing Nox4 protein synthesis within the time frame of our experiments as cytoplasmic staining did not differ between thick ascending limbs in the no‐flow and flow groups. Newly synthesized Nox4 would have to traverse the rough endoplasmic reticulum and likely the Golgi for proper folding and trafficking. Thus, de novo synthesis should result in an increase in cytoplasmic expression in addition to nuclear expression unless all of the newly synthesized protein makes it to the nucleus within 20 min, which seems unlikely. Additionally, the fraction of Nox4 in the nucleus is relatively small compared to other regions. It seems unlikely that the Nox4 gene would be transcribed for such a short period of time to only cause such a minor increase in total expression as would be required to explain the increase in nuclear Nox4. Finally, changes in protein localization in response to different stimuli are not uncommon. Previous studies of our group reported the flow‐induced translocation of nitric oxide synthase 3 (NOS3) from the cytoplasm to the apical membrane of thick ascending limbs (Ortiz et al. 2004). Others studies have reported Nox4 translocation from the cytosol to the plasma membrane (von Lohneysen et al. 2010) and nucleus (Xiao et al. 2009; Guida et al. 2013) in different experimental models.

We next addressed whether Nox4 is first activated by flow then translocates to the nucleus or vice versa. To do this we first studied the effect of PKC inhibition, because flow‐induced, Nox4‐dependent O_2_^−^ production requires PKC activation. For this subset of experiments, we used the general PKC inhibitor staurosporine at a 10 nmol/L dose, but because this drug fluoresces at the same wavelength as DHE, Gö 6976, a PKCɑ/*β* specific inhibitor, was used instead when we measured flow‐induced O_2_^−^ production in the presence of a PKC inhibitor. Inhibiting PKC blocked both flow‐induced translocation of Nox4 to the nuclear region and O_2_^−^ production. To our knowledge, there are no studies reporting Nox4 phosphorylation, but several reports have shown that Nox4‐dependent O_2_^−^ synthesis is PKC‐dependent. PKC inhibition reduced O_2_^−^ by 80% in thick ascending limbs of diabetic rats (Yang et al. 2010) and prevented flow‐induced O_2_^−^ production (Hong et al. 2010). Additionally, stimulation of PKC enhanced O_2_^−^ production (Hong et al. 2010). However, since PKC inhibition blocked both flow‐induced Nox4 translocation and O_2_^−^ production, these data provide no information as to which process comes first.

Since many translocation events require an intact cytoskeleton, we next studied whether disrupting the actin cytoskeleton would provide additional information. Our data show that the disruption of the actin cytoskeleton prevents Nox4 recruitment to the cell nucleus; however, it did not block the increase in O_2_^−^ production. These data suggest that activation of Nox4 comes before translocation to the nucleus.

The dependence of Nox4 translocation on an intact cytoskeleton is similar to previous reports. Integrity of the actin cytoskeleton is necessary for NOS3 translocation (Ortiz et al. 2004). However, in this case translocation occurs before activation as disrupting the actin cytoskeleton also blocked NO production. Similarly, the cytoskeleton is required for NHE 1 exchanger activity in thick ascending limbs (Watts et al. 2005) likely due to trafficking of the transporter to the basolateral membrane.

Poldip2 has been reported to bind and increase the activity of Nox4. Whether Poldip2 is expressed and its localization in thick ascending limbs was heretofore unknown. We found that Poldip2 was localized within many compartments in thick ascending limb cells similar to Nox4. Exposure of tubules to luminal flow did not alter Poldip2 expression in the nucleus. This result is contrary to our initial hypothesis, but is in agreement with the study from Lyle et al. (2009) where they reported that in the absence of Poldip2, Nox4 no longer localized in focal adhesions, but it was still present in the nucleus of vascular smooth muscle cells. Binding to Poldip2 may ensure proper Nox4 cytoplasmic localization, as suggested by the coincident patterns, but it is not required for Nox4 translocation. Furthermore, since Nox4 activation occurs independently from its translocation, Poldip2 could be contributing to Nox4 activity, as suggested by the high degree of colocalization of both proteins in the cytoplasmic region. Further studies are required to elucidate this relationship.

We conclude that increasing luminal flow leads to Nox4 translocation to the nucleus in a PKC‐ and cytoskeleton‐dependent manner. However, Nox4 translocation is not required for flow‐induced increase in O_2_^−^ production. Translocation of Nox4 is independent from Poldip2. Thus, O_2_^−^‐dependent changes in transcription in thick ascending limbs likely require Nox4 translocation to the nucleus.

## Conflict of Interest

None declared.
